# Examining intersectional inequalities in access to health (enabling) resources in disadvantaged communities in Scotland: advancing the participatory paradigm

**DOI:** 10.1186/s12939-018-0797-x

**Published:** 2018-09-24

**Authors:** Anuj Kapilashrami, Sara Marsden

**Affiliations:** Global Health Policy Unit, University of Edinburgh, Edinburgh, UK

## Abstract

**Background:**

Multiple structural, contextual and individual factors determine social disadvantage and affect health experience. There is limited understanding, however, of how this complex system works to shape access to health enabling resources (HER), especially for most marginalised or hard-to-reach populations. As a result, planning continues to be bereft of voices and lived realities of those in the margins. This paper reports on key findings and experience of a participatory action research (PAR) that aimed to deepen understanding of how multiple disadvantages (and structures of oppression) interact to produce difference in access to resources affecting well-being in disadvantaged communities in Edinburgh.

**Methods:**

An innovative approach combining intersectionality and PAR was adopted and operationalised in three overlapping phases. A preparatory phase helped establish relationships with participant groups and policy stakeholders, and challenge assumptions underlying the study design. Field-work and analysis was conducted iteratively in two phases: with a range of participants working in policy and community roles (or ‘bridge’ populations), followed by residents of one Edinburgh locality with relatively high levels of deprivation (As measured by the Scottish Index of Multiple Deprivation, a geographically-based indicator. See http://www.gov.scot/Topics/Statistics/SIMD/DataAnalysis/SPconstituencyprofile/EdinburghNorthern-Leith). Traditional qualitative methods (interviews, focus groups) alongside participatory methods (health resource mapping, spider-grams, photovoice) were employed to facilitate action-oriented knowledge production among multiply disadvantaged groups.

**Results:**

There was considerable agreement across groups and communities as to what healthful living (in general) means. This entailed a combination of material, environmental, socio-cultural and affective resources including: a sense of belonging and of purpose, feeling valued, self-esteem, safe/secure housing, reliable income, and access to responsive and sensitive health care when needed. Differences emerge in the value placed by people at different social locations on these resources. The conditions/aspects of their living environment that affected their access to and ability to translate these resources into improved health also appeared to vary with social location.

**Conclusion:**

Integrating intersectionality with PAR enables the generation of a fuller understanding of disparities in the distribution of, and access to, HER, notably from the standpoint of those excluded from mainstream policy and planning processes. Employing an intersectionality lens helped illuminate links between individual subjectivities and wider social structures and power relations. PAR on the other hand offered the potential to engage multiply disadvantaged groups in a process to collectively build local knowledge for action to develop healthier communities and towards positive community-led social change.

## Background

Health inequalities research in the UK has made significant progress in offering explanations for the systematic differences in health that exist between different population groups. This body of work generally focuses on distribution of health by social class or socio-economic position and employs quantitative/experimental study designs. The latter disregards lived experiential accounts of health inequalities. Moreover, the privileging of socioeconomic position undermines other aspects of social location and marginality [1] (gender, ethnicity, dis/ability, etc.). We know, however, that multiple structural, contextual and individual factors determine social disadvantage and affect health experience. Graham ([2], p. 3) accounts for these multiple disadvantages in offering a normative conception of health inequalities as: ‘systematic differences in the health of people occupying unequal positions in society’.

Recent literature points to the relevance of an intersectionality approach to understanding what these systematic differences are and the pathways through which these are established. However, there is little consensus on the most appropriate methodologies to gain an understanding of how this complex system works and how such analysis can be enriched by voices of those at the margins of the system or the ‘hard-to-reach’ groups. The People’s Health Manifesto for Scotland,^1^ developed using action research, began to explore how policy and politics can be informed by community voices [3]. This experience informs this research, but reliance on third sector (i.e. voluntary & non-profit) groups, practitioners, academics and advocates limited our reach of voices from the margins- those that are multiply disadvantaged. Methods to address this complexity thus remain under-developed.

Accounting for these gaps in research and policy debates, this paper reports findings of a study that aimed at operationalising intersectionality by combining it with PAR to gain insights into perceptions and lived experiences of disadvantaged communities. This approach was employed to deepen understanding of how multiple factors interact to produce difference in access to material, environmental, socio-cultural and affective resources that affect well-being in disadvantaged communities.

The paper is structured in three parts: First, we situate the study in key strengths and gaps in existing literature on Health Enabling Resources (HER) and health inequalities. Second, we describe our methodological approach including a description of methods and participants alongside a detailed account of the process adopted. The discussion offers a critical reflection on both process and findings, addressing the question of what this combined approach can add to our understanding of how multiple disadvantages generate differences in access to resources affecting well-being.

### Health enabling resource (HER)

There is no agreed definition of HER in the literature. Public health literature is particularly deficient and ambiguous in defining what resources people regard as health(y) or (un)healthy. Epidemiological research pays disproportionate attention to lifestyle choices and adoption of healthy behaviours, and to a lesser extent on the environments restricting these choices [4]. The area of “enabling environments remain(s) under-theorised and under-researched” ([5], p. 388). Some health inequality scholars with a social determinant focus, have paid attention to enabling ‘neighborhoods’ [6] spaces [7] and environment [8]. This body of work emphasises social capital and how it shapes access to HERs, and offers a conception of HER as “including healthcare services, sources of healthier food options, and public recreation spaces” [9]. Here, the notion of ‘place’ (or neighbourhood) offers some useful insights.

Dominated largely by epidemiological and sociological research, interest in ‘place’ has been triggered by an impetus on studying the effects of local context on individual health and well-being. While there is a longer tradition of studying variation in health status across local and regional contexts, the new generation of research on place and health can be distinguished for attending to the multilevel causation of these contextual differences [10].

Neighbourhoods and health literature emphasizes the effects and influences that a place may exert on health outcomes and associated behaviours of resident populations. Galster [11] identifies four key domains or mechanisms of such influence: *social interactive* including social cohesion and networks; *environmental* including both physical assets/infrastructure and exposure to substances; *geographical* pertaining to particular macro political-economic conditions such as investments and redevelopment; and *institutional* mechanisms that may enhance vulnerability through stigmatization, financing etc. While this body of literature contributes to our understanding of socio-spatial patterning in health outcomes, it has been criticized for offering a “partial account for inequalities in health across local contexts” [10, 12]. The criticisms rest on two main grounds: First, methodologically, overwhelming use of cross-sectional design limits a nuanced historical account of how neighbourhoods have developed over time, and an understanding of the broader macro-level socio-economic processes (e.g. restructuring, migration) that mediate people’s relationship with place over time. The second ground relates to the absence of a relational perspective and the importance of informing this work with ideas of ‘place making’. Critics argue that ‘place’ is not merely an assemblage of material elements but socially (re)produced through human interaction; and this *social* and *relational* production of place affects material outcomes including health [13, 14]. Such place-making draws on diverse resources, those that exist in the setting itself, and those that result from the practices and the relations that individuals and groups develop with, and in, the place. In this context, as Duff [5] argues, places may be enabling to the extent that they make available specific resources and facilitate the production and circulation of those resources that are necessary for realising specific enabling practices and processes.

To this critique we add another omission in studies that offer neighbourhood explanations for inequalities. These have tended not to consider differential distribution of resources within a defined geography and how social location of multiply disadvantaged groups residing in the same ‘place’ may have different access to available resources; thereby producing differential effects on health and well-being. For instance, while a neighborhood health service may be open to all residents, specific cultural, informational, and social barriers may prevent specific groups – such as first generation Black and Asian Minority Ethnic (BAME) communities with language barriers or those with stigmatized livelihoods such as prostitution and behaviours such as drug use – from using it.

In sociological studies, HER has been understood as those resources that support, to a varying extent, three distinct functions: “the maintenance (and improvement) of health and well-being, the mitigation of specific risks and vulnerabilities, and creation of health promoting or enabling places” [5]. Cameron Duff [5] identifies three categories of resources in a framework applied to the context of harm reduction:

*Material* encompassing economic, functional and practical assets that are fundamental to enabling health promoting practices. They may include direct services or health products (life-saving drugs or advice and information) as well as the capacity to utilize it via stable income, education, and transport among others.

*Social* resources refer to the myriad relationships and processes that characterize social life, and support creation and maintenance of personal networks. These include “relations of trust and reciprocity associated with social capital”, as well as relational attributes that support building of social cohesion, which in turn impacts health by facilitating participation, leverage and access to resources [9].

*Affective* resources have been understood as a combination of “discrete feeling states” that are expressed in everyday life and “action potential” [15] or their agency to affect and be affected by their interaction with material and social environment. These interactions may either enhance individual’s power to act or diminish it.

This paper contributes to this body of research by illustrating differences in ‘local’ perceptions of HER and barriers to access faced by multiply disadvantaged groups in one site in Edinburgh.

The study did not have pre-determined assumptions around HER. Instead, in alignment with the fundamental basis of Participatory Action Research (PAR), our aim was for these understandings to emerge from the participants to build insight into HER from the different (disadvantaged) standpoints. Thus, analysis was an inductive iterative process informed by data from participants and supported by Duff’s framework of categorizing resources.

In presenting local perceptions, we seek to illustrate the value for health inequalities research in employing an intersectionality lens to research design and analysis, operationalised through a participatory action paradigm to reach marginalised populations. Specific questions answered in corresponding sections of this paper are:

What resources do participants find health enabling? What, if any, are the differences in access to HER for people occupying different social locations?

### Description of area

The focus for the study was communities in and around the Leith end of the busy Leith Walk, a main thoroughfare connecting Edinburgh with its port area of Leith, an area hit hard by past deindustrialization (closing of shipping, whaling and whisky industries), severe economic decline and present-day austerity. As a result, like many docks around the UK, it became synonymous with crime, violence and prostitution. The area straddles, and is integral to, two of the 32 City of Edinburgh council wards (Leith and Leith Walk) with a population of approximately 60,000 people [16]. It has changed massively in the last 20 years and is now characterized by a high density of housing (flats) and population, greater mix of population from outside Scotland than the rest of the city, and a higher working age population [17]. Yet, the area is characterized by high inequalities, parts of it among the 20% of most deprived areas in Scotland (SMID), and with an above city-average proportion of people dependent on benefits [18].

## Methods

This qualitative study employed an intersectionality lens and used PAR to illuminate links between individual subjectivities and wider social structures [19]. We sought to engage participants, particularly those who are marginalized by mainstream health policy and planning, to collectively build local knowledge for action to develop healthier communities [20]. Ethics clearance was obtained through the Ethics Committee of the University of Edinburgh.

***Intersectionality*** has been variously employed by social scientists, as both an analytical framework and methodology, to understand “the multiple interacting influences of social location, identity and historical oppression” on experience of inequalities [1]. Its growing popularity in inequalities research is attributed to wider recognition that the experiences of these social locations need to be understood, not as separate dimensions of inequalities (gender, ethnicity, class, disability) but as greater than the sum of their parts [19, 21]. That said, application of intersectionality in health inequalities research is underdeveloped, limited to experiences of specific disadvantaged groups or greater attention to the ‘holy trinity’ of race, gender and class [22].

***Participatory Action Research*** (PAR) is an approach to inequalities research, distinctive for its collective orientation and emancipatory action-potential. It involves a collective and reflexive inquiry process that researchers and participants undertake to better understand and improve upon the processes and conditions under investigation [23]. As an expression of “new paradigm science” [24] PAR resists the idea of participants as ‘subjects’ to be researched, advocating for their active involvement in collective analysis and co-creation of local knowledge. While PAR is increasingly utilized to study inequalities, its application to capture multiple dimensions of disadvantage that affect health has received much less attention.

This methodological framework brought an emphasis to designing an approach that explored individual lived realities and endeavoured to maximise participants’ control over the production of knowledge. For example, we stressed participants’ autonomy over defining HER, choosing appropriate methods from a wider range of participatory methods, adapted to participants’ needs. Besides standard qualitative research techniques, such as exploratory and semi-structured interviews, the following participatory methods were used to explore and analyse local knowledge, including supporting action-oriented analysis:

- *Participatory mapping*: an interactive and deliberative method used to represent spatial knowledge of local communities. Participants engage in an analytical process by creating a visual representation of their environment in relation to a social issue [25].
- *Spider-gram* to explore as a group (purposively recruited to have shared social situations or conditions) what they think “counts” as “health enabling resources” in (or near) their community, and a discussion about how it is distributed.
- *Photovoice*: an image-based method involving a “process by which people can identify, represent and enhance their community” [26] through photographs that capture their surroundings/ experiences, and through ongoing reflection on their learning around their personal struggles and community concerns. Each group was given an orientation meeting followed by two meetings to discuss photographs, adapting the precise formulation to the needs of different groups. For instance, *photovoice walks* were organised for participants lacking capacity to plan or commit to sustained participation, for a variety of reasons related to chaotic lives, mental health problems etc. The walks allowed a group process to emerge but also flexibility for individual photography without disrupting group activity.

### Process

The research was undertaken in three overlapping phases and we reviewed and developed our approach as the work progressed.

### Phase I preparatory phase

A nine-month *preparatory phase* helped establish relationships with participant groups and policy stakeholders, including public health practitioners and representatives of third sector (i.e. voluntary & non-profit organisations), NHS Lothian and the Council. One-to-one interviews with four NHS and Council representatives, and six third sector representatives, generated a critical understanding of the current local context (demographics, council reorganization, policy trends etc.), helped confront researcher assumptions, and informed the selection of sites.

From this the researchers mapped out local public and third sector services, identified key informants, and helped facilitate/participated in consultative processes undertaken by the Council (such as on Health and Social Care, social isolation among others). The latter gave us key understandings of formal mechanisms of consultation and how policy decisions might be made. It also laid the foundations for facilitating better access to those processes for local populations as research progressed. “Snowballing” from the key informants, we moved into the *field-work and analysis* stage, conducted iteratively with a range of participants over nine months.

### Phases II & III knowledge generation

A key challenge to designing inequalities research is reaching those most marginalized by the structural determinants of interest. To help overcome the tendency of research to capture experiences of those who are accessible; we adopted a deliberative multi-phase strategy (outlined below) giving attention to a range of entry points used to access participants.

### Phase II: Bridging participants

Third and public sector initiatives, identified and shortlisted in Phase I, provided introductions to local population groups, including those pre-defined by specific needs (care, health information/advice), identity or axes of marginalisation (BAME/faith-based, homeless) or by their use of a range of services, social activities, and skills development initiatives (e.g. digital skills) intended to tackle social disadvantage.

We engaged “bridge” participants – community workers and volunteers associated with delivering these initiatives - through a range of methods: Ten in-depth interviews, four resource mapping and spidergram groups (of 4–5 participants), and two photovoice groups (4 participants each). As well as generating knowledge themselves (on wider economic changes and HER), they also assisted in identifying and working with local populations.

### Phase III: Resident participants

Emphasising those voices that are less well-heard, we recruited participants with experience of homelessness, precarious incomes and poverty, and food insecurity often combined with substance use. To understand how relative privileges and disadvantages were distributed in the community, we ensured population groups represented mix of age, ethnicity (Scottish/ British majority, ethnic minority) and gender. Most groups were dominated either by men, or by women offering us rich gendered understandings of differences.

Methods were centered around two activities: one-off HER mapping and more sustained, focused photovoice work. Participatory mapping was carried out in pre-formed groups of (mostly) residents and a group of BAME school pupils.

The following are the resident groups which participated, labelled as referred to subsequently in this paper:

i. Elderly British women (Skills exchange): 4 users of a local mutual help scheme, mainly British/Scottish over 60, limited financial means (Resource mapping)
ii. Men’s group (homeless): 5–7 ad hoc attendees at social / food group. Mixed age(30–65) with experience of, or at risk of homelessness, all white British/Scottish. (Resource mapping, photovoice walk)
iii. Women’s support (chronic health): 7 Members of a peer support group for women with long-term health conditions - mixed age 20–65), all white European, mostly British/Scottish (Spidergram)
iv. Under15 girls (BAME): 5 secondary school pupils from BAME communities, mainly Muslim and Sikh but mixed nationalities. (Resource mapping, photovoice)
v. Elderly BAME women: 8 women, all except two over 60 and first generation immigrants who came to Edinburgh/UK after marriage (Resource mapping).
vi. Elderly Scottish women (crafts group): 8 members of a craft group, all women over 70 (mainly 80s), low-income (Resource mapping).
vii. Support group (low-income parents): 5 women on low and/or insecure income, all white British/Scottish, aged 30–45 (Photovoice).
viii. Breakfast (homeless & food insecure): weekly breakfast drop-in for homeless people relying on food banks, and in many cases using drugs or alcohol– mixed group in terms of gender and age. Mostly white British/Scottish. (Observations and Brief interviews/discussions with volunteers and 5 among 30 attendees)

## Results

### Perceptions of HER

Here we follow Duff’s classification of enabling resources into material, social and affective. As Duff argues, most resources are not so straightforwardly categorised. They may be material in nature but critical for social connections, and affecting individual’s emotional state. This notwithstanding, the above categories serve as a starting point to describe resources identified by participants, and the enabling and disabling practices and environments these generate. Note that because the nature of the engagement with the Breakfast (homeless) group was more limited, any references to it are explicit. Thus, reports of “all groups” or “most groups” are not inclusive of the Breakfast group unless specified.

### Material resources

Material resources came mainly in tangible forms like healthcare services, food, clothing, income (including benefits), safe warm housing, transport, green spaces, faith organisation facilities, such as meeting spaces, kitchens, etc. but also in intangible forms such as advice and information, and time availability (and associated stress).

All groups indicated that *healthcare services* of one sort or another were beneficial to their health. All groups specifically identified GP surgery, while some identified it along with a local treatment centre providing various outpatient services, notably physiotherapy, pain management, and an ADHD clinic for children. Other health services noted were dentists and pharmacists. While all participants identified services as serving an important function to maintain good health, only the older BAME group identified ‘health information’ as an important resource along with services.

Identification of services as a resource came with complaints of long waiting and short consultation times from the Women’s Support (health), Craft, & BAME Groups.

[We need] *more time in the* [GP] *surgery. More time for counselling. Faster referrals.*

*There should be more clinics open for depression. Only Junction is accessed by everyone. The younger generation is suffering more with depression. They need help, support to get there, back and forth. When you need to speak to somebody you need to do so soon, but referral takes between 6 to 12 weeks. –* Elderly BAME women

Along with the community workers, this was identified as a problem of inadequate numbers of GPs in the area. Community workers in all groups were critical of health services rationed regardless of patient need.

All but two groups of participants identified specific *public/community resources* – libraries, places of worship (local church, mosque and temple) and a community centre. The reasons were varied, with different resource benefits to different groups. The local library was valued for information (books and IT), free WiFi access, support with IT, free warm area to wait (with children), friendly staff, and some social activity. The outliers - Men’s group (homeless) and BAME groups – despite awareness of their existence and noting specific benefits they may offer to others, did not regard these as enabling their health (though the young women did note the benefits of the books and information provided by their school library).

*Physical exercise and urban spaces* that facilitate this were cited by all groups, though the way they benefited from this varied considerably. The Men’s group (homeless) considered gym (and swimming) as a potential resource but, instead emphasised aesthetics of their urban environment that encouraged them to walk. The Under15 BAME women valued walking more as a break from school and as a form of relaxation; other groups identified generally indoor activities that were mostly facilitated by (mostly ad-hoc initiatives of) the third-sector. Likewise, all groups identified green spaces (such as parks) as a HER though its use was limited to a few groups, for instance, the men’s group (homeless) as part of their walking options, or low-income Support group and Under15 BAME girls for sitting, socialising and relaxing.

*Many of us are diabetic. Lot of the ladies have been told by their GPs that they need to walk for their arthritis. So, exercise is very important for our health.* - Elderly BAME women.

*Public amenities* such as the roads and transport services emerged as a resource appreciated across all groups. The benefit to participants with free bus passes (over 60, under 16, with certain disabilities) seemed particularly striking, extending their mobility and expanding their access to other resources significantly.

*Without my bus pass I would be housebound.* - Men’s group (homelessness).

*Well, we’re always doing something. And in* [the sheltered housing], *when they see us going out again arm in arm - they’ll say from their window, that’s that two away again. We’re just going out the building to go on the bus. [...] And we like to go on the tram. And when we get to the end we go out that door, walk up the platform and come back again. [laughing] And the wee lassie that’s coming in the wheel chair - she loves it because the trams have got big windows right down and there’s a place for the wheelchair and she can go in -and she can see everything that’s going on.* - Elderly Scottish women

Where perhaps the bus service for these groups enabled their access to resources, these did not emerge significant for BAME women participants, in part due to restricted movement but also not having money for tickets.

*Food and good diet* was identified in various ways. The men’s group (homelessness) also put social spaces and food together on their map, and the need for a good diet was expressed with general agreement that they could improve their own diets, often reliant on cheap takeaway food. That said, they identified affordable take-aways as a health resource, partly for convenience (when out) and partly because of a lack of skills or inclination to cook. The elderly Scottish women’s group noted the availability of supermarkets locally as being a good thing for their access to good food. A young couple at the Breakfast group saw access to a kitchen at their homeless hostel as important for keeping a healthy diet. The younger BAME women, aside from easily being able to pick up sandwiches for lunch and sweets for their breaks, mentioned food in the context of socialising with family and close friends.

In the group sessions, foodbanks were mentioned as a resource though only in the context of their availability at places of worship (one local church and a Sikh temple) with no one disclosing their own need for them except on one occasion during the men’s photovoice walk. At this instance, one participant mentioned how much he had gained from several services (including a housing association, local faith communities (Sikh and Christian)) when he had been homeless and hungry:

*I wouldn’t be where I am today without this organisation [housing association]* […] *the Sikh Temple, they do a lot for the community and they feed the homeless, and yes including me before now.*

Men’s group (homelessness) group (photovoice walk participant).

Most groups mentioned the importance of *good housing* where they felt safe, well and warm. This was mentioned alongside the importance of a good and safe neighbourhood, both in terms of helpful and friendly immediate neighbours and enhancement of the use of local amenities such as parks.

*Income* was explicitly identified as essential to take advantage of many resources, including social and affective resources (e.g. by the Craft group of elderly women). This was mentioned in several resident groups but discussed in depth by community workers. As well as a basic level of income, the certainty of income was also considered important to reduce levels of stress associated with a fear of lack of money for basic living costs. In this context, workers identified job centre and employment opportunities as a resource, with both enabling and disabling potentials explored later in this paper.

### Social resources

As with material resources, many of the social resources identified by participants (e.g. social space such as café) have aspects of both the material and affective, but are listed here for their central social purpose.

All groups identified some form of social resource as health enabling. A variety of social spaces were noted, either where social activities were organised (e.g. weekly social drop-in for men, craft activity groups, bowling, sports, yoga etc) or where they could meet friends (e.g. cafés, parks or group outings to the beach). Generally, having something to do, including social outings was valued for the social connection it enabled. A participant of the Women’s support (health group) expressed feelings of isolation and loneliness outside these groups, “I sometimes feel lost on a Sunday because there’s no one else around*”.* The value placed on these social activities were most significant among BAME women. For example, young women moving to Edinburgh/ the UK after marriage (with deficient social networks and strong social control on their movement) or elderly BAME women who were house-bound due to child-care responsibilities and a culture of not occupying public spaces on their own. The excerpt below highlights the importance of these social connections (maintained through periodic activities) for their well-being.

*P4: I suffer from depression a lot. Become ill this time of the year for 3–5 months. But coming here and meeting friends means a lot. We go out as a group and that changes my mood. Even my kids have noticed the difference. Say you should go out more. I feel ashamed to. Sometimes you feel they don’t want to know you because you are a widow.*

*R: Why? Have you felt stigmatised elsewhere?*

*P4: I never go on my own anywhere. It’s only this group we come to.*

*P5: If this group was not there, we will just be in the house, looking after grandkids.*

Elderly BAME women

As well as social, such events also provide affective resources, such as help “to get out of the house” and some sort of structure in one’s day. The Craft group (as well as community workers) identified an intergenerational project with local schoolchildren as a resource that offered both learning (e.g. digital skills) but also facilitated social contact with children.

Whilst social networks of place were generally found to be good for health this was not always so for those struggling with addiction. This was noted by community workers, and also by a Breakfast (homeless) participant struggling with alcohol addiction, whose friends used his house to take drugs and “paid” him in alcohol: *“I need to get out of Leith”.*

The opportunity to volunteer and to give ‘back’ to the community was viewed as an important social resource across all groups. This took a variety of forms, such as knitting for babies (by the Elderly Craft group), cooking food for homeless (BAME women), and exchanging affordable clothing at charity shops (Support group (parents)). These roles offered participants a sense of purpose, enhancing their self-esteem, including reducing any sense of dependency. For BAME women, who felt excluded and less integrated, this also served as a pathway to supporting other marginalised and deprived communities (such as homeless or those relying on food banks).

*We knitted something like 50–60 of these.. scarves and hats and various things, But what I think they [women] enjoyed most was that they had gone from being people in the community who get looked after, and have things going wrong - to people who are contributing for other people. They’re all in their 80s and 90s anyway. Probably some of the people who benefited from these were younger than them, but they were happy.*

– Elderly woman, Crafts group.

### Affective resource

Note that the way we use this resource category does not precisely follow Duff’s [5] typology. He emphasises affective resources as precisely *not* “feeling-states”, but rather the resources external to people that directly impact/create those feeling states (that in turn determine an individual’s capacity to act). However, in many instances, feeling-states are described as resources. For example, confidence was identified as an important HER, as was a sense of belonging. We therefore use this category to report and discuss both feeling states and the resources that Duff [5] would consider to be “affective resources”.

These feeling-states were often discussed in depth amongst the community worker participants, with one notable discussion on what constituted resilience, feeling-states of hope, etc. as well as affective resources, such as structure and routine.

*CW1: If they’re that type of person, who’ve got good resilience, that’s a health enhancing resource.*

*CW2: But routine structure, that is more important.*

*CW1:Having a purpose, or volunteering. Feeling valued.*

*CW3: Yeh, it all comes back to self-awareness, worth.*

*CW1: Sometimes people can be resilient to an extent but then, they’re able to ask for help. That’s a really good skill to have.*

*Having confidence* to take part in a group, to ask for help or services was deemed essential for benefiting from many resources, enabling social connections, and while interacting with health and social care systems/bureaucracies. Confidence was gained through being part of a group, having a shared identity (as identified by resident groups), as well as being supported/accompanied (e.g. at their first attendance at a service such as job centre). Lack of confidence was also associated with notions of self-esteem and worth and, notably, material resources such as clothing, income and appearance. A community worker pointed to the “sense of embarrassment that people experienced by not being able to meet unexpected expenses whilst participating.”

*Trust* of workers delivering services too was identified as a key resource enabling use of those services, through for instance, longer term relationships with individual workers reducing the fear of being refused help or losing support they have come to rely on. This – particularly among worker groups – was a recurring theme, where long-term funding was identified as crucial for enabling workers to build trust amongst service users in both those individuals and in continuity of the service. Factors reported as impeding the building of trust included Funding cutbacks, short term funding for projects, and the awarding of contracts to commercial organisations, displacing local providers with strong community connections reported as impeding it). In contrast to negative encounters with public services (explored later), people reported friendly interactions and sense of gratitude with staff at the local treatment centre.

*It* [Treatment Centre] *does wonderful things for the community. Staff are very community orientated and when you go into there, they’re very kind in how they treat you […] There’s been times where you don’t get that treatment in certain* [healthcare] *places but for some reason the community here starts from hospital and they are with the community.*

Men’s group (homelessness) (photovoice walk participant).

A *sense of belonging* either to place or group was expressed as a resource. The Men’s group expressed pride at belonging to the Leith area, and each pre-formed group valued belonging to their group; with Women’s craft group enthused about how they had made it more than just crafts (outings, and helping others). Community workers echoed this view, that a sense of belonging was important:

*Place and belonging like, a lot of people in Leith, they just love it - it’s got its history, its identity and that actually makes them feel good about where they live, shared common identity which can actually make them feel, y’know, that really helps. Belonging to groups as well [as place], people feel that gives purpose. “Aw, we’re a proper group now. We do things. we help each other, we achieve things”*.

Community Worker

Linked to the pride in Leith was a perception (in non-BAME groups) that the community, on the whole, valued diversity relatively highly. The Men’s group suggested this on the photovoice walk, community workers echoed this, and the Skills Exchange group put it on their map as a resource that expanded personal horizons. BAME women did not express a sense of belonging in relation to Leith, which could be attributed to their limited mobility (and other factors), but placed value on elements of diversity (for e.g. specific provisions -Asian clothes, grocery stores) in the area that catered to their needs.

### Factors shaping uptake of resources

**Fig. 1:**
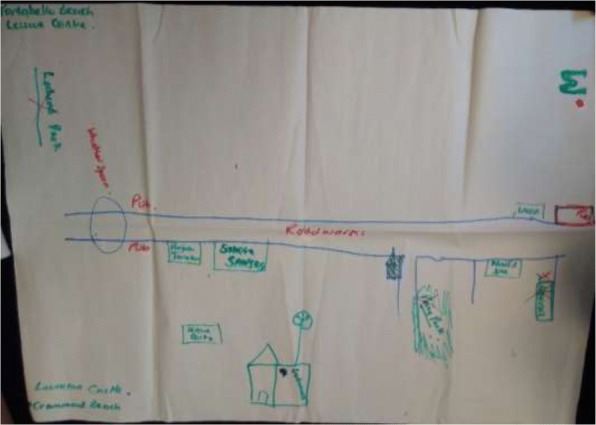
Resources map: Older BAME women

**Fig. 2:**
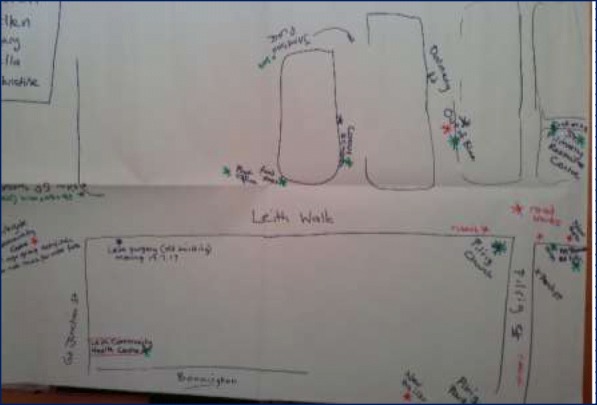
Resource map: Older Scottish women

**Fig. 3:**
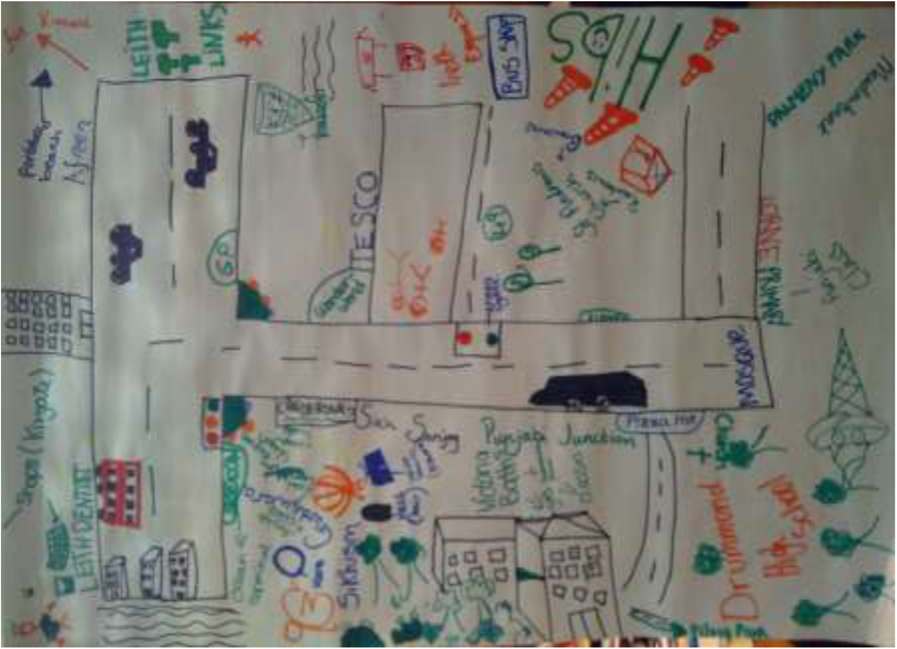
Resource map: Under15 girls (BAME)

Although often the same resources are identified by the groups as health enabling, significant differences were observed in the richness of resource maps generated of the area and what mix of resources people at different social locations access to achieve healthful living. These differences were most notable along race/ ethnicity, gender, poverty and age lines. Figures 1 and 2 contrasts perceptions of older BAME women and older Scottish women. While aspects of physical environment (parks and places to worship) were common in both, very few community resources and public services (including health care) were seen as health enabling by BAME women. Figure 3, which captures perceptions of second and third generation BAME girls growing up in Edinburgh, appears in stark contrast to the older BAME women, illustrating a richer and enabling view of the same place.

Differences were also prominent in factors shaping the uptake of resources, and their deployment in creating practices that enable or disable health. We present these under institutional, geographical, and environmental pathways/ influences [11].

### Institutional

Among ethnic minorities, difficulties in access to healthcare services were compounded by differences in health beliefs and values, as well as communication barriers that limited the health enhancing potential of health care services.

*Health issues in our community are really hidden. No one wants to share information, even to the doctor, especially the older generation.*

– BAME women (elderly)

*It’s a stigma. You don’t want to say that you’re ill. I see a lot of folk saying that they’ll cope. And they are at risk of neglect because they can’t just say we need help […] We’ve someone with dementia –their daughter took a very long time to admit because she was blaming herself- “did I not take care, did I not listen to her?”*

*–* Staff of a BAME health inclusion service

Interestingly, while language was a barrier for some, members of a BAME organisation expressed anger at the mismatch between needs and provisions, linking it with ignorance among public providers/ planners that leads to institutional racism.

*The biggest thing for me is that they do not seem to have understood the difference between British Asians and Immigrants. We are not immigrants. We are the children/ grandchildren of immigrants. We took our kids to school. We could speak English no problem* [*...*] [the problem is] *making assumptions; someone running away to find an interpreter without even speaking to you, just on the way you’re dressed.*

There was a notable consistency among all groups of working age in how the interface with survival-critical welfare services were experienced as demeaning, anxiety provoking, and discriminating against marginalised groups. Several had experienced extended disputes entailing hours of time spent making a case and the frustrations of a perceived lack of logic, sometimes with a catastrophic impact on an individual’s income.

Changing circumstances trigger changes in benefits and proof requirements. Those on varying incomes, likely for many precarious workers and for parents working around school holidays, are most likely then to experience frequent delays in benefits, with the risk of rent arrears. The health support group discussed this at length pointing out that they have to prove their income every month, and as one participant expressed “*It is a nightmare getting benefits”.* This was also striking in the experience of retaining homeless status (and the associated duty of the council to rehouse). Those deemed homeless must make three bids a week to an online index of available housing in the City. This group has less access to the internet either because of a lack of skills or resources, and although they receive some support to do this, what should be quick online tasks (e.g. to update contact details) become much more cumbersome via an intermediary. This system also increases the likelihood of them having to move away from their local area and existing social networks. This system for housing allocation also affects elderly in social housing who need to move if their accommodation is no longer suitable. Many do not have the required digital skills, and often no access e.g. mobility, sight and no one with responsibility to help.

A striking example of impact of such institutional discrimination on feeling-state was “brown-envelope phobia” describing the fear and dread of what is contained in a letter from an authority, usually regarding entitlement (or not) to benefits (Women’s support (low income)).

### Environmental – physical assets & exposure to harm

Although public amenities, green spaces and facilities were identified as a potential resource (to enable physically active living), their use was constrained for participating groups, albeit for different reasons. These included cost/ affordability, and feeling out of place, for instance at the gym:

*The thing is you gotta pay. Not only that, if you go there, there’s also these like, Adonises, ken like? If you go there with your wee cheap trainers and aw’ that, you feel - it’s no like going to the gym when you were younger. There’s too much posing and aw’ that. Gym bunnies we call them! They’re just addicted to going to the gym*.

Men’s group (homelessness)

Another notable constraint was feelings of fear and anxiety that impact on participants’ mobility:

*Alleyways are really dark. When you come down this long alley, there is a gate. I have seen people there, swapping things. So, safety is a concern. I wouldn’t go, wouldn’t let my kids either*.

Elderly BAME women

The older BAME women identified two leisure centres in their HER map but reported not using them for cultural and religious reasons (of sharing space with men).

*But one big issue in Edinburgh for women from our background is that there are no women-only sessions in pools or gyms and leisure centres. Only one is the Victoria leisure centre with one 45 mins session for women. And it is always full.*

- Elderly BAME women

Impeding people’s ability to move around safely and easily, there was frustration with dumping of waste/ furniture on the roads, endless roadworks, the unwelcome decision to place cycle tracks on pavements, and the lack of benches for less mobile people to be able to walk with rest breaks. The Men’s group (homeless) also criticised the reduction in provision of (free) public toilets as it hindered their free movement in the urban landscape. Participants also expressed the parks and green walkways being tucked away in the city with better access for those in less deprived areas than Leith.

On eating healthily, community workers and the Men’s group, identified lack of cooking skills as preventing people from consuming healthy foods. The Men’s group commented on how cheap and convenient takeaway food can be in their physical environment, and for one with substantially restricted mobility (and needing to use a walking stick), having to carry food from the supermarket on foot, reduced the amount he cooked for himself. Exposure to unhealthy foods emerged, on probing, among older BAME women who stressed eating habits (time, food) and intergenerational changes:

*I do worry about fast food. Because they are so easily accessible, order on phone. And our children rely a lot on these. If you don’t find food at home, then just dial in a pizza*.

- Elderly BAME women.

### Geographical & structural

Underlying these barriers to access were structural determinants of poverty, worsened by an austerity agenda. The most notable dis-affective resource was the stress of not having a reliable income and the constant grind of having to watch every penny. This arose in several settings though most openly discussed by community workers who have witnessed the stress it puts on people, especially families. Interactions with the welfare system further exacerbates this stress, as explained under institutional pathways. Poor job prospects, lack of income and gendered dimension of poverty also emerged in discussions with BAME organisations, mostly impacting 2nd or 3rd generation women.

*The biggest thing is, we don’t have any money. We’ve got women (second generation or even third) who were dropped out, married very young, who are now in their 40s need to go back to work. They lack confidence, not because of language but lack of understanding, skill, educational attainment. Nobody’s acknowledging the fact that settled migrant communities just as others are facing issues of employment, training, education.*

- Elderly BAME women.

A recurrent theme was the rapidly changing urban environment and community, to the detriment mainly of social capital. Reasons identified included the gentrification processes, increased holiday lets, expensive unaffordable cafes, social housing policies and a transient population. Younger generations have been forced out and this has, with high rental turnover, reduced support networks from family and long-term neighbours. There was a critical discussion in the low-income women’s support group, as well as among community workers about how social spaces had become commercialised and there was nowhere to go without pressure to buy something, significantly limiting the social spaces they could access.

Related to this changing social fabric, most expressed concern about increased social isolation and the negative effects on their health (anxiety, stress and fear) from, difficult neighbours, with drug dealing, addiction, theft, gang intimidation all being mentioned. Lives were adjusted to ensure safety, for instance, Craft and Women’s support groups members not going out after dark. This was also relevant to Leith as a whole, with all groups (including the men’s group) identifying some pubs as increasing their fear of crime. Given the lack of a strong sense of belonging to Leith, such fears were most pronounced among BAME women:

*At our end, I live in [name of street], we have got teenagers burning bins, fire engines coming all the time. That’s because pubs allowing them to get alcohol at such a young age. Pubs here are a negative influence on our health. Even while parking I’ve to be very careful. I like to dial someone if I have to pass huddles of people smoking, drinking on the streets. I don’t feel safe.*

## Discussion

A particular tendency in explanations for socio-spatial health inequalities has been to either focus primarily on the damaging aspects (i.e. distribution of risks and exposure) of social and physical environments and geographies; or treat ‘risk’ and enabling environments as two distinct and opposing entities. Less is known about the enabling characteristics of place i.e. resources that people draw on to maintain (or enhance) their health, and the extent to which these generate or impede healthy practices.

Addressing these empirical and methodological gaps, we operationalized an approach to understand experiences of HER from the standpoint of those located at the intersections of multiple disadvantages. Findings presented above focus on both distribution of resources in a place, as well as capacities and constraints on populations to use these for improved health.

Reflections on findings: Claiming resources and the practices of place-making.

A range of resources were identified as necessary for realizing health across all participating groups residing in Leith. Adopting Duff’s typology, we identified these as material, social and affective resources, although attention was paid to capturing the dynamic interaction between these and participants, and how their use/non-use shaped people’s relationship with the area.

Clear consensus emerged across groups on material resources such as reliable income, access to culturally sensitive and responsive healthcare services in the region, non-discriminating welfare services, secure housing and safe neighbourhood, as well as wider access to opportunities to build social connections and networks. In addition, a range of affective resources were identified from sense of belonging and of purpose, feeling valued, and confidence and self-esteem to make use of the material and social resources available in the region.

Commonalities in understanding of HER aside, clear patterns of difference emerged among people at different social locations in what resources they use, what practice these enable and, to a lesser extent, how such ‘enabling’ is associated with improved health. Results are telling of how differential aspects of their social location and living environment (e.g. poor living conditions and chaotic lifestyles) impinge on their capacities and agencies to translate availability of resources into healthy practices. Here, contrary to the dominant understandings of inequalities, differences were not patterned along socio-economic position or ethnicity or gender. Instead, what emerged was how multiple disadvantages interacted to limit knowledge of HER in the area and affected individuals’ capacity to benefit from these. For instance, intersections of gender, ethnicity and age along with poverty, longstanding health conditions (including mental health) shaped older women’s agency to access green spaces and other community provisions for physical activity.

While proximity to, or distribution of these material resources was important, a range of preconditions and pathways were necessary to facilitate their health enabling function i.e. their use by populations to enable health-promoting practices. Health inequalities research highlights specific social mechanisms/processes as having a critical role in this process [27, 28]. In this study, we identified three key pathways of influence – institutional, structural and environmental- that mediate such claim-making and place-making process. While successful negotiation of resources demands considerable resilience, understanding of entitlements and assertiveness, changes in physical environment, macro-structures and institutions impede this process. For instance, re-development/gentrification in Leith (expensive cafes, housing), changes in benefit systems, and lack of diversity-friendly services increased social isolation, pushing multiply disadvantaged groups deeper into adversity and limiting their reach of material resources. Furthermore, interface with critical public welfare services (housing, employment) exacerbated discrimination, resulting in distress and anxiety, most severely experienced by those at intersections of poverty, precariat employment, chronic (mental health) problems, insecure housing/homelessness and low education levels. This was striking in the case of social housing arrangements, which radically re-shape place-making. The artificial requirement for the homeless to make three bids per week can force the most vulnerable into an area of the city they have no connection with.

Leith is characterised by a rich diversity of resources, including vibrant third sector initiatives identified as having the potential to disrupt the conversion of adversity to poor health (by offering material resources and enhancing social capital via initiatives identified in resource maps). However, we identified ways in which institutional arrangements are not responsive to actual community needs and actually reduced a place’s supply of HERs and access to them. Initiatives ending due to short-term funding (resulting from cut-backs) destroys trust (through for example, lost connections between individual service users and staff, and suspicion of future services’ lack of reliability), and initiatives with narrow remits limit trusted workers’ ability to support vulnerable people who will be less likely to approach an unknown person (thus wasting trust as a resource). The short-term funding of similar services over 30+ years suggests also that institutional arrangements are not adapting to long-term needs (e.g. resources for one-time immigrant communities, now settled). Awarding contracts to remote organisations (in a bid to reduce cost) has dismantled networks of local community workers and volunteers, a major loss of social (and affective) resources.

### Reflections on the process

The epistemological coherence of Intersectionality and PAR have been emphasised by many scholars [29]. Yet, to-date, scholarship on health inequalities lacks a systematic attempt to explicitly bring these together.^2^ Hence, amid calls for deeper exploration and extension of intersectionality using mature methodological approaches [30], we aimed to operationalise an approach that attempted to bring intersectionality to inform our methodology and analysis of disadvantages, and PAR into our research *praxis* and relationships.

Adopting PAR allowed us to engage resident populations in a critical reflexive inquiry that has triggered wider processes of planning and social action, involving community groups, link workers and key policy networks. For instance, organisations that acted as ‘bridge’ have taken active interest in hosting photo(voice) exhibits and planning citizens’ tribunal to build an action agenda on health inequalities. Engaging key representatives of policy and planning agencies (such as Edinburgh Council and NHS Lothian) from the outset also extended spaces for participatory dialogue to include those who were previously excluded (and remained in the periphery). Currently, the researchers and key participants across sectors (including council, academia, service delivery (health and community development)) are discussing several opportunities/media to strengthen policy and practice links (via for e.g. Scotland’s National Action Plan for Human Rights, Equality networks among others). In doing so, a major contribution of PAR has been to promote collaborative peer to peer interactions, in an environment where power is more dispersed and shared, to build shared collective identities through the critical analysis of their own social location, agency and changes they wish to see.

Alongside generating a nuanced understanding of how multiple structures of inequalities (ethnicity, homelessness, insecure or no job, and gender) determine access to and use of HER, the process also enabled community workers’ reflections on who are excluded from their programmes and discussion on how these gaps be best addressed. Intersectionality informed both our level of analysis and recruitment strategy. The latter prevented reification of social groupings, encouraging focus on social dynamics (processes, structures and institutions) rather than one social category or axes of inequality/ vulnerability. By focusing on groups that represented multiple structures of disadvantage, and in some contexts relative privileges, rich insights on inter- and intra- ‘group’ differences emerged. For example, a more positive relationship with place was observed among young girls of BAME origin as compared to the older first generation immigrant women who had limited awareness of available resources, and limited opportunity (restricted to one social group) in using these to enable healthy practices.

As would be expected, some of the most marginalized people we worked with were those experiencing multiple and varying compounding disadvantages. For some this resulted in chaotic lives and a low capacity to sustain attention beyond short-term survival (e.g. those attending free breakfast services were homeless, without employment, dependent on food banks, and often using drugs and other substances such as alcohol), and so enabling their contribution required careful consideration of ethics and adaptation of methods. Collaborating with community partners (or bridge participants) with compatible aims of reaching the most marginalized, was key. Introductions to potential participants (many with reason to be suspicious of strangers and with little hope for change), by trusted people in trusted spaces made the work possible. However, limited funding and time constraints implied that very few of the most disenfranchised populations who are not reached by a service-public or third sector- could be reached for a sustained period. These limitations notwithstanding, it is noteworthy that while the research was time bound, the mobilization process it set off and critical consciousness it engendered among third sector ‘bridge’ populations is currently informing longer term processes (for example citizens tribunal via the People’s Health Movement) that are aiming to reach other stigmatised and excluded populations.

## Conclusion

By identifying a spectrum of HER that multiply disadvantaged populations can draw on and illuminating aspects of their material and psychosocial environment that prevent their uptake, the study offers useful pointers to effective strategies for improving reach of health resources.

Much epidemiological research (and political party agendas on health inequalities) emphasises individual level factors and “irresponsible lifestyle ‘choices’” [[30], p. 3] as explanations for poor health. As a departure from this view, a focus on enabling environments, allows for examining individual experiences in relation to the wider *institutional*, *structural* and *environmental* influences that determine health.

In this context, PAR and social mobilisation that it entails, helps populations gain self-esteem and critical consciousness of both their privileges and oppressions, identify common cause and begin to challenge unquestioned developments and resulting inequalities. Alongside working with policy planners from the outset, such social mobilisation can bring about incremental changes and catalyse greater commitment to the pursuit of socially inclusive and just development.

## Abbreviations

BAME: Black Asian Minority Ethnic
CW: Community worker
HER: Health Enabling Resources
PAR: Participatory Action Research

## Footnotes

1 The Scottish People’s Health Manifesto can be found here: The process through which the manifesto was developed is described in Kapilashrami et al. [1].

2 A notable exception is an empirical study by Reid et al. [29] that explores the mutuality of intersectional analysis and Feminist Participatory Action Health Research in the Hankivsky (Ed) volume on Health Inequities in Canada. Besides, several other scholars have made a case for their contribution in developing this to strengthen a feminist post-postcolonial research agenda.
